# Nutrigenomic Effect of Hydroxytyrosol in Vascular Endothelial Cells: A Transcriptomic Profile Analysis

**DOI:** 10.3390/nu13113990

**Published:** 2021-11-09

**Authors:** Maria Annunziata Carluccio, Rosanna Martinelli, Marika Massaro, Nadia Calabriso, Egeria Scoditti, Michele Maffia, Tiziano Verri, Valentina Gatta, Raffaele De Caterina

**Affiliations:** 1 National Research Council (CNR) Institute of Clinical Physiology (IFC), Campus Ecotekne, Via Monteroni, 73100 Lecce, Italy; marika.massaro@ifc.cnr.it (M.M.); nadia.calabriso@ifc.cnr.it (N.C.); egeria.scoditti@ifc.cnr.it (E.S.); 2 Department of Medicine, Surgery and Dentistry “Scuola Medica Salernitana”, University of Salerno, Via Salvador Allende, 84081 Baronissi, Salerno, Italy; rmartinelli@unisa.it; 3 Department of Biological and Environmental Science and Technology (DISTEBA), Campus Ecotekne, University of Salento, Via Monteroni, 73100 Lecce, Italy; michele.maffia@unisalento.it (M.M.); tiziano.verri@unisalento.it (T.V.); 4 Department of Psychological, Health and Territorial Sciences, School of Medicine and Health Sciences, “G. d’Annunzio” University, 66100 Chieti, Italy; v.gatta@unich.it; 5 Cardiology Division, Pisa University Hospital, 56124 Pisa, Italy

**Keywords:** hydroxytyrosol, nutrigenomics, gene expression, transcriptomics, microarray analysis, endothelial cells, endothelial dysfunction, olive oil polyphenols

## Abstract

Hydroxytyrosol (HT), a peculiar olive and olive oil phenolic antioxidant, plays a significant role in the endothelial and cardiovascular protection associated with olive oil consumption. However, studies examining the effects of HT on the whole-genome expression of endothelial cells, which are prominent targets for vasculo-protective effects of olive oil polyphenols, have been lacking. This study aims to comprehensively evaluate the genomic effects exerted by HT, at the transcriptional level, in endothelial cells under resting or proinflammatory conditions. Human umbilical vein endothelial cells (HUVECs) were treated with 10 µmol/L HT for 1 h and then stimulated with 5 ng/mL interleukin (IL)-1β for 3 h. Total RNA was extracted, and gene expression profile assessed with microarray analysis. Functional enrichment analysis and pathway analysis were performed by Ingenuity Pathways Analysis. Microarray data were validated by qRT-PCR. Fixing a significance threshold at 1.5-fold change, HT affected the expression of 708 and 599 genes, respectively, in HUVECs under resting and IL-1β-stimulated conditions; among these, 190 were common to both conditions. Unfolded protein response (UPR) and endoplasmic reticulum stress resulted from the two top canonical pathways common between HT and HT-IL-1β affected genes. IL-17F/A signaling was found in the top canonical pathways of HT modified genes under resting unstimulated conditions, whereas cardiac hypertrophy signaling was identified among the pathways affected by HT-IL-1β. The transcriptomic analysis allowed pinpointing immunological, inflammatory, proliferative, and metabolic-related pathways as the most affected by HT in endothelial cells. It also revealed previously unsuspected genes and related gene pathways affected by HT, thus broadening our knowledge of its biological properties. The unbiased identification of novel genes regulated by HT improves our understanding of mechanisms by which olive oil prevents or attenuates inflammatory diseases and identifies new genes to be enquired as potential contributors to the inter-individual variation in response to functional food consumption.

## 1. Introduction

The Mediterranean diet is recognized as a cornerstone of chronic disease prevention. Adherence to a Mediterranean diet style reduces the risk of cardiovascular and neurodegenerative diseases and even premature death overall [1,2,3]. Extra virgin olive oil is a key symbol of the Mediterranean diet. Obtained directly from olives by mechanical extraction, it represents, at the same time, the primary source of fat as well as of health-promoting components, particularly polyphenols, with effects that principally include a reduced risk of atherosclerosis and related cardio-cerebro-vascular sequelae [4,5]. Hydroxytyrosol (HT, 3,4-dihydroxyphenylethanol) is the most prominent phenolic constituent of olives, olive oil, and their byproducts, such as olive mill wastewater. It is appreciated for its antioxidant, anti-inflammatory, and antiatherogenic properties. Its strong antioxidant actions are due to radical scavenging and to the gene expression and activation of antioxidant enzymes, which in turn guarantee a reduced lipid peroxidation of low-density lipoprotein (LDL) cholesterol [6]. Crucial supportive studies demonstrated that minor components of olive oil, particularly HT and derivatives, reduce atherosclerotic risk factors, improve plasma lipid levels, and repair oxidative damage related to cardiovascular diseases [7]. Of note, the European Food Safety Authority (EFSA) released a declaration regarding the benefits derived from the daily ingestion of 5 mg of olive oil polyphenols (HT and conjugated forms) on the protection of LDL from oxidation, highlighting how suitable intake of natural functional food components can reduce the plasma levels of oxidized-LDL, a crucial cardiovascular risk factor [8]. Oxidized-LDL supports foam cell formation, stimulates monocytes chemotaxis, induces the adhesion of circulating blood monocytes to vascular endothelium, the earliest obliged and critical step in atherogenesis [9]. Current research indicates that, besides reducing oxidative stress markers and atherosclerotic risk factors, olive oil polyphenols induce beneficial changes in the expression profile of genes involved in atherosclerosis, vascular inflammation, and oxidative stress [10]. Human nutritional intervention studies comparing similar olive oils with different polyphenol contents pointed out the nutrigenomic effects of olive oil polyphenols in cardiovascular disease prevention. In a randomized crossover trial [11], gene expression microarray analysis on peripheral blood mononuclear cells (PBMCs) isolated from patients with metabolic syndrome revealed that a breakfast based on virgin olive oil, high in polyphenols (398 ppm), repressed the expression of proinflammatory genes, linked to atherosclerosis, obesity, dyslipidemia, and type 2 diabetes mellitus when compared with common olive oil (low in polyphenols, 70 ppm) [11]. Concordantly, microarray reports indicated that, in healthy volunteers, an acute or chronic intake of virgin olive oil induced changes in gene expression related to insulin resistance, oxidative stress, and inflammation [10]. Following the results from human intervention trials, in vitro studies using cell model systems relevant to cardiovascular diseases, such as vascular endothelial cells and monocytes/macrophages, reinforce the nutrigenomic effects of olive oil polyphenols as a mechanism for their vasculo-protective role [12].

The endothelium, the thin layer of cells that lines the interior of blood vessels, plays a vital role in maintaining homeostasis over the entire vascular system. Endothelial cells can regulate vascular tone, permeability, coagulation, and inflammation [13]. However, vascular injuries derived from several triggers, including cardiovascular risk factors, oxidative stress, and chronic inflammation, can lead to endothelial activation and dysfunction, resulting in disturbance or loss of normal endothelial functions [14]. Part of the known health protective effects of the Mediterranean diet has been linked to improvements in endothelial function, acting on different vascular territories in the presence of several vascular risk factors. Human evidence has indeed been accumulated regarding the beneficial modulation by the Mediterranean diet or extra virgin olive oil of microvascular endothelial function assessed as ischemic reactive hyperemia, as well as of the macrovascular response of peripheral and central arteries in terms of flow-mediated vasodilation and carotid intima-media thickness, a marker of subclinical atherosclerosis, respectively [15,16]. These effects could potentially be translated into a reduction in cardiovascular events and are conveyed by different cellular and molecular mechanisms, including epigenetic regulation, increase in the number of endothelial progenitor cells, an index of endothelial regenerative capacity, or improvements of nitric oxide availability, a potent endothelial vasodilator, and of markers of endothelial activation and inflammation [15].

Our research group has previously shown that olive oil polyphenols directly influence the vascular wall protecting the endothelium from the effects of proinflammatory and proatherosclerotic stimuli [17]. In an in vitro model of early atherogenesis consisting of cultured human umbilical vein endothelial cells (HUVECs) activated by proinflammatory and proatherosclerotic triggers, we have previously found that olive oil polyphenols with antioxidant activity, including HT, significantly inhibited events connected with endothelial activation, along with the expression of adhesion molecules such as VCAM-1, E-selectin and, to a lesser extent, ICAM-1, after stimulation with virtually any stimulus able to elicit the coordinated expression of such genes [17,18,19]. This effect was accompanied by a functional counterpart, i.e., reduced monocyte adhesion to cytokine-activated endothelium [19]. Moreover, our findings showed that olive oil polyphenols, at nutritionally relevant concentrations, inhibited inflammation and endothelial dysfunction by down-regulating the gene expression of matrix metalloproteinase and proinflammatory enzymes [17,18,19,20,21,22,23,24,25]. These effects are mediated by the inactivation of the redox-sensitive transcription factors, including nuclear factor kappa B (NF-κB) and activator protein-1 (AP-1), which regulate the concerted expression of inflammatory genes. Although several studies have demonstrated that HT may positively affect endothelial dysfunction, a comprehensive evaluation of the endothelial genomic effects is lacking. Indeed, up to now, only studies that adopted a targeted approach, i.e., analyzed changes in the expression of specific targets, have been performed in HT treated endothelial cells.

In recent years, the growth of omics technologies, by simultaneously comparing thousands of genes, has offered the opportunity to extensively investigate pathophysiological mechanisms involved in a variety of human diseases, including atherosclerosis, as well as to learn more about the molecular and metabolic effects of external interventions, including nutraceuticals, in a fashion not biased or restricted by a priori hypotheses [26]. In an attempt to deepen the interactions between nutrients and gene expression, high-throughput gene expression using microarrays is applied to analyze transcriptomes. The discovery of several noncoding RNAs with regulatory functions emphasizes the study of transcriptomics as an end-point of regulatory control in the molecular mechanisms of nutrients [27,28,29]. Since the transcriptome varies according to cell type and environmental conditions and considering the importance of transcriptomics to identify unexpected, early, and novel effects of the dietetic bioactive molecule, our study aimed to assess the effects of HT on human endothelial cells, which are prominent targets for vasculo-protective effects of olive oil polyphenols, under resting and inflammatory conditions, as mimicked by the cytokine interleukin 1 beta (IL-1β) stimulation. A transcriptomic approach was exploited to identify major genes and pathways modulated by HT that may be responsible for its vasculo-protective effects.

## 2. Materials and Methods

### 2.1. Materials

HT (≥98% purity) was obtained from Cayman Chemical (Ann Arbor, MI, USA) and interleukin-1β (IL-1β) from Sigma-Aldrich (St. Louis, MO, USA). Cell cultures materials were obtained from Gibco/BRL (Life Technologies Italia Monza MB, Italy). Unless otherwise indicated, all other reagents were purchased from Sigma-Aldrich (St. Louis, MO, USA).

### 2.2. Cell Culture and Treatment

HUVECs were isolated from segments of discarded umbilical cords from normal-term deliveries and treated anonymously, conforming to the principles outlined in the Declaration of Helsinki after specific permission was granted by the local health authority. HUVECs were cultured in Medium 199 as previously [30] and used up to the fifth passage from primary culture. For treatment, confluent HUVECs were treated with 10 μmol/L HT for 1 h and then stimulated with IL-1β (0 or 5 ng/mL) for an additional 3 h. Cellular toxicity was checked by trypan blue exclusion and MTT (3-(4,5-dimethylthiazolyl-2)-2,5-diphenyltetrazolium bromide) assays. All cultures featured a cobblestone cell morphology and positive staining for von Willebrand factor, as previously reported [30].

### 2.3. Experimental Design and RNA Isolation and Analysis

HUVECs were treated with 10 μmol/L HT for 1 h and then stimulated with IL-1β (0 or 5 ng/mL) for 3 h, after which the cells were collected and total RNA extracted using the Qiagen RNeasy kit (Qiagen, Milan, Italy) following the manufacturer’s instructions. The total RNA was stored at −80 °C until use. We determined the concentration and purity of RNA by NanoDrop ND-1000 UV-Vis Spectrophotometry (NanoDrop Technologies, Wilmington, DE, USA) and the integrity of RNA by using the Agilent 2100 Bioanalyzer (Agilent Technologies Inc., Santa Clara, CA, USA). The same RNAs were used for microarray and quantitative reverse-transcription polymerase chain reaction (qRT-PCR) analysis. In addition, we performed qRT-PCR analyses on different HUVEC samples to further confirm microarray data results. No variation in the total RNA yield was observed under the different experimental conditions tested (data not shown).

### 2.4. Microarray Analysis

For microarray analysis, RNAs were labelled, and hybridization was performed using the Gene Expression Hybridization kit (Agilent Technologies Inc., Santa Clara, CA, USA) as previously described [31]. Briefly, total RNA from HUVECs treated with HT, IL-1β or HT-IL-1β and HUVEC untreated controls (CTR) was amplified using cyanine-3/cyanine-5 labelled CTP with Agilent low RNA Input Fluorescent Linear Amplification kit (Agilent Technologies), according to the manufacturer’s protocol. Equal amounts of cRNA’s from HUVEC control (labelled with Cy3) and HUVECs treated with HT, IL-1β or HT-IL-1β (labelled with Cy5) were mixed together and hybridized. The slides were washed, dried, stabilized, and then scanned with the Agilent’s dual-laser microarray scanner (G2565AA), and the image data were processed using Agilent Feature extraction software. This software calculates log ratios and p-values for valid features on each array and provides a confidence measure of differential gene expression performing outlier removal and background subtraction. Gene expression profiles were generated using the 4 × 44 K glass slide Whole Human Genome Oligo Microarray G4112A (Agilent Technologies). For each sample, the technical replicates were performed. Each array assessed total RNAs from treated endothelial cells (HT, IL-1β or HT-IL-1β) compared with RNA obtained from control endothelial cells (untreated endothelial cells). Raw data were processed by the GeneSpring 10 software (Agilent Technologies), as previously described [32]. Statistical analysis was performed using background-corrected mean signal intensities from each dye channel. Microarray data were normalized using intensity-dependent global normalization (LOWESS). Differentially expressed RNAs were identified using filtering by the Benjamini and Hochberg False Discovery Rate (FDR) (*p*-value < 0.05) to minimize the selection of false positives. Differentially expressed genes (DEGs), with expression levels greater than 1.5-fold increase or 1.5-fold decrease compared to the controls, were used for further analysis.

### 2.5. Gene Ontology and Pathway Analysis of Transcriptomic Data

Gene ontology (GO, http://www.geneontology.org/ (accessed on 3 November 2021)) analysis was carried out to determine the functional annotation of the DEGs identified and the biological domain with respect to three aspects: biological process, molecular function, and cellular component.

Functional enrichment and pathway analysis of DEGs were performed using Ingenuity Pathways Analysis (IPA) 8.0 (Ingenuity® Systems, http://www.ingenuity.com (accessed on 3 November 2021)) by using the core analysis function of IPA. Functional enrichment analysis was based on up- and down-regulated genes to identify the biological processes and functions over-represented in the examined gene lists. Canonical pathways analysis identified the pathways most significant for the input data set. The significance of the association between the data set and the canonical pathway was determined based on two parameters: (1) a ratio of the number of genes from the data set that map to the pathway divided by the total number of genes that map to the canonical pathway; and (2) a *p*-value calculated using Fischer’s exact test, determining the probability that the association between the genes in the data set and the canonical pathway is due to chance alone. In addition, based on the information stored in the Ingenuity^®^ Knowledge Base, upstream regulator analysis was performed using the IPA software, which is based on the examination of the known targets of each transcription regulator in the list of DEGs, comparing their direction of change to what is expected from the literature. The prediction algorithm calculates a z-score, and it is designed to reduce the chance that random data would generate significant predictions.

### 2.6. qRT-PCR of Candidate Target Genes

To validate microarray data, qRT-PCR was performed on the same samples used for microarrays experiments and on additional samples obtained under the same experimental conditions. Total RNA (1 μg) was converted into first-strand cDNA using the High-Capacity cDNA Reverse Transcription Kit (Applied Biosystems, Monza, Italy). The qRT-PCR was performed in the Bio-Rad Biosystems CFX384 Touch Real-Time PCR Detection System using SYBR Green PCR Master Mix. All reactions were carried out in triplicate on three independent sets of cDNA. The human cDNA fragments were amplified using primers synthesized by ThermoFisher (ThermoFisher Scientific, Rodano, Italy) and reported in Table 1. We explored the expression of the following genes: HERPUD1, DNAJB9, HSPA5, APLN, LTB, CCL2, and DGAT2. To account for possible variations related to cDNA input or the presence of PCR inhibitors, the endogenous reference genes glyceraldehyde 3 phosphate dehydrogenase (GAPDH) and 18S ribosomal RNA (18S rRNA) were simultaneously quantified in each sample, and the data were normalized accordingly.

### 2.7. Statistical Analysis

Raw data were processed with the GeneSpring 10 software (Agilent Technologies), and DEGs were identified using the Benjamini and Hochberg False Discovery Rate (FDR), with a *p*-value for significance set at 0.05. Student’s *t*-test for paired observations was used for comparisons of qRT-PCR results. Data are expressed as fold change. Deregulated genes were defined as fold change > 1.5. *p*-values < 0.05 were accepted to indicate statistically significant differences.

## 3. Results

### 3.1. Gene Expression Profiling of HT Treated Endothelial Cells

**Figure 1:**
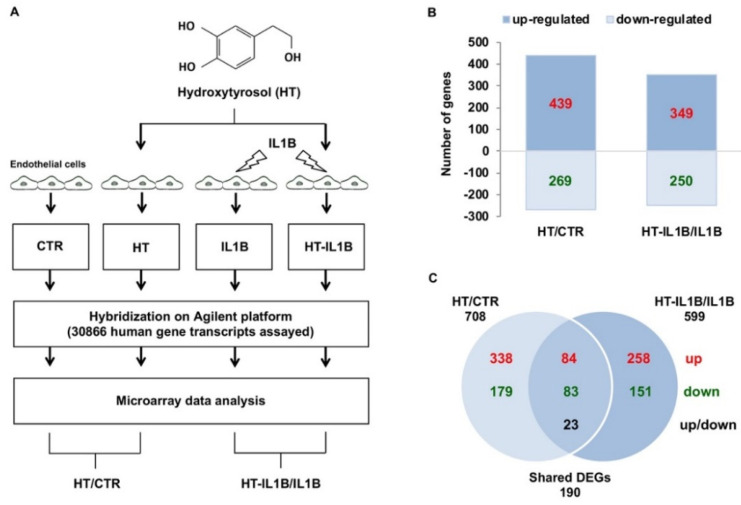
Differentially expressed genes in response to HT treatment in endothelial cells under basal and inflamed conditions. (**A**) Experimental design: HUVECs were treated with 10 μmol/L HT for 1 h and then stimulated with/without 5 ng/mL IL-1β for an additional 3 h, after which the total RNA was extracted, and microarray analysis was performed. (**B**) The bar diagram indicates the number of differentially expressed genes (DEGs), which resulted in up-regulated or down-regulated genes in the HT vs. control (HT/CTR) and the HT plus IL-1β vs. IL-1β (HT-IL1B/IL1B). (**C**) Venn diagram reporting the numbers of unique and shared DEGs (up-regulated or down-regulated) in HT/CTR and HT-IL1B/IL1B. In red up-regulated DEGs, in green down-regulated DEGs, in black DEGs with opposite regulation (up or down).

Total RNA was isolated from tripled cultures of HUVECs incubated with 10 μmol/L HT for 1 h and subsequently stimulated with the proinflammatory cytokine interleukin 1 beta (IL-1β) at 5 ng/mL for another 3 h, together with their respective untreated controls (Figure 1A). Cellular cDNA was prepared and processed for hybridization to the human oligonucleotide DNA microarray. To identify differentially expressed genes (DEGs) as a factor of treatment, pairwise comparisons were made between HT-treated cells and untreated control cells (HT/CTR) or between HT plus IL-1β and IL-1β stimulated cells (HT-IL1B/IL1B) (Figure 1).

By analyzing the microarray data, we found that, among the total of 44K gene probes, HT deeply affected endothelial gene expression deregulating the expression of 708 genes with respect to control endothelial cells (2.3%) (Figure 1B and Table S1). Of these genes, 439 were up-regulated, and 269 were down-regulated (Figure 1B). HT treatment before IL-1β stimulation modified the expression by about 25% of IL-1β-deregulated genes. In particular, HT pretreatment altered the expression of 599 genes, of which 349 genes were up-regulated and 250 down-regulated (Figure 1B). Moreover, 190 DEGs were shared by HT treated endothelial cells both at baseline and at IL-1β stimulated conditions. Many shared DEGs were modulated in the same direction (84 up-regulated and 83 down-regulated), and 23 shared DEGs exhibited opposite regulation (Figure 1C).

**Figure 2:**
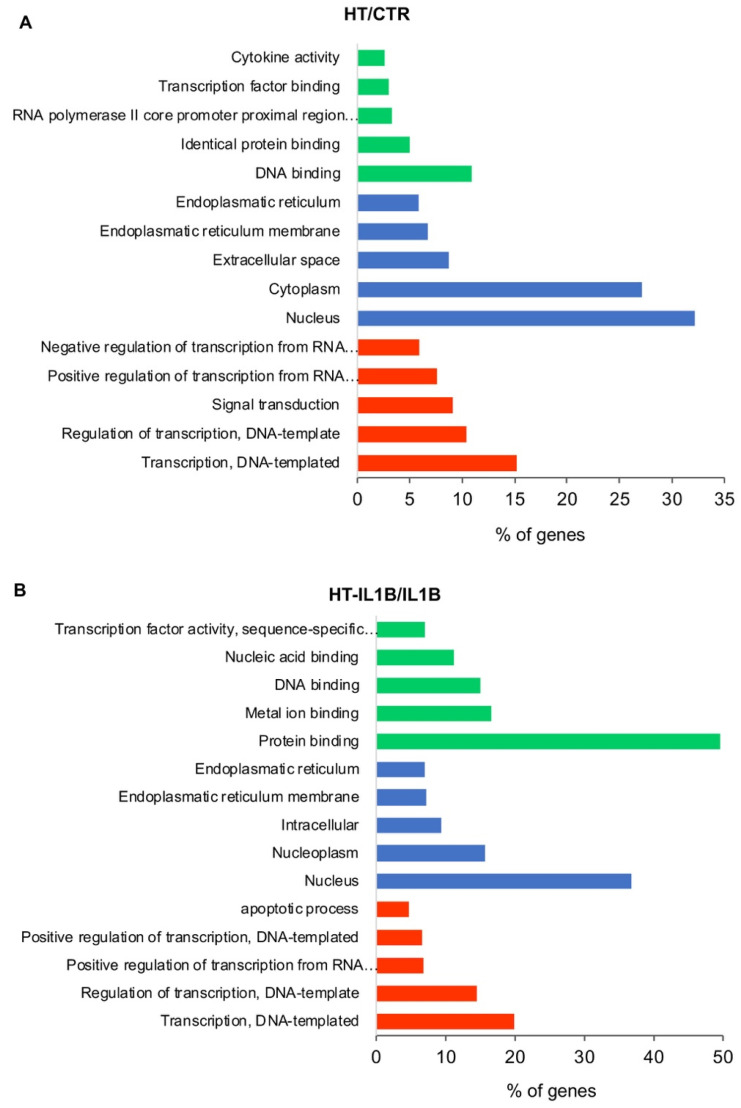
GO functional classification of the DEGs. The distributions are summarized in three main categories: biological process, molecular function, and cellular component. The *x*-axis indicates the percentage of involved genes/total genes, and the *y*-axis indicates the top five gene function subclasses in HT/CTR (**A**) and HT-IL1B/IL1B (**B**).

To analyze the basic features of gene expressions in endothelial cells, we first used the freely available tool, Gene Ontology (GO). Figure 2 shows the three basic classes in HT treated endothelial cells, i.e., “Biological process”, “Molecular function”, and “Cellular component”. In each class, the top five gene function subclasses with the highest ratio of significantly regulated genes are listed out.

Among biological processes, “Transcription, DNA-templated”, “Regulation of transcription, DNA-templated” and “Positive regulation of transcription from RNA polymerase II promoter” were significantly perturbed both by HT (Figure 2A) and HT-IL-1β treatment (Figure 2B), which suggest for HT the ability to interfere with gene transcription regulation. Correspondently, HT altered the expression of 76 and 81 genes coding for transcription factors, which regulated gene transcription at basal and inflammatory conditions, respectively. According to these data, among the cellular components, the “Nucleus” was the subclass most influenced by HT treatment both under control and IL-1β stimulated conditions, followed by “Endoplasmatic reticulum membrane” and “Endoplasmatic reticulum” (Figure 2). The top activated molecular functions included “DNA binding”, “Identical protein binding”, “Transcription factor binding” and “Cytokine activity” for HT; and “Protein binding”, Metal ion binding”, “DNA binding”, “Nucleic acid binding” for HT-IL1B (Figure 2).

To further analyze the microarray data, the lists of identified DEGs were imported into the IPA software, which mapped 469 genes (Table S1) of the total 708 genes modified by HT at resting conditions (HT/CTR) and 435 genes (Table S2) of the total 599 genes altered by HT under inflamed conditions (HT-IL1B) (Table 2). As shown in Table 2, comparing the signals of HT treated endothelial cells with control endothelial cells, the results revealed that the expression of 71 genes was altered significantly by equal or greater than 2-fold, whereas the expressions of 398 genes were modified between 1.5-fold and 2-fold.

The analysis of genes affected by HT under inflammatory conditions (HT-IL1B) revealed that the expressions of 45 genes were perturbed significantly by equal or greater than 2-fold, whereas the expressions of 390 genes were modified between 1.5-fold and 2-fold (Table 2). Overall, HT treatment, both at basal and inflammatory conditions, induced large fold changes in a few individual genes and minor changes in a more extensive gene list.

The top ten significantly altered genes in HT/CTR and HT-IL1B/IL1B with a fold change of at least two are tabulated in Table 3 and Table 4, respectively. The top significantly up-regulated genes were Homocysteine-Responsive Endoplasmic Reticulum-Resident Ubiquitin-Like Domain Member 1 (HERPUD1) and DnaJ Heat Shock Protein Family, Hsp40, Member B9 (DNAJB9), with 6.603- and 4.179-fold change in HT/CTR (Table 3) and 5.383- and 4.232-fold change in HT-IL1B/IL1B, respectively (Table 4).

**Figure 3:**
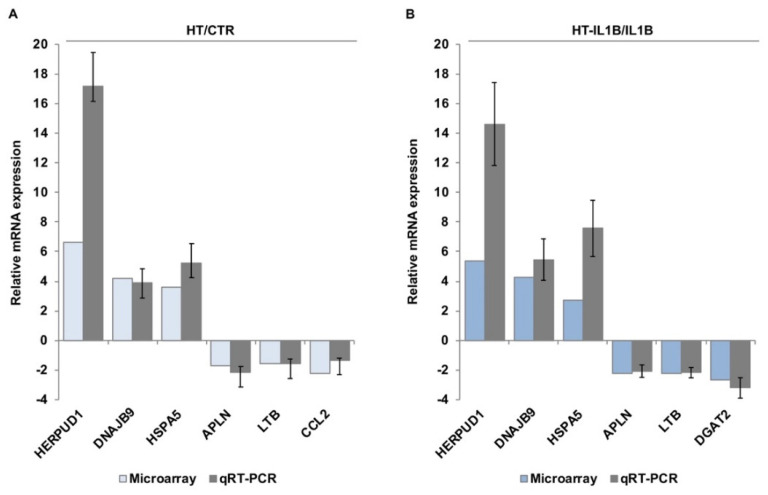
Validation of microarray data by qRT-PCR. HUVECs were treated with/without 10 μmol/L hydroxytyrosol (HT) for 1 h (**A**) and then stimulated with 5 ng/mL IL-1β (IL1B) for an additional 3 h (**B**). qRT-PCR was performed for HERPUD1, DNAJB9, HSPA5 (up-regulated) and APLN, LTB, CCL2 and DGAT2 (down-regulated). The results were expressed as the relative mRNA expression. Each value represents the mean ± SD of three separate experiments. Microarray-based relative mRNA expression was also shown for comparison.

To validate the accuracy of the microarray data, some representative genes with the highest significance levels and expression ratio or genes belonging to top canonical pathways were selected, and their expression levels were confirmed by qRT-PCR. Based on this criterion, we selected the following genes for validation: HERPUD1 (Homocysteine-responsive endoplasmic reticulum-resident ubiquitin-like domain member 1), DNAJB9 (DnaJ Heat Shock Protein Family, Hsp40, Member B9), HSPA5 (Heat Shock Protein Family A, Hsp70, Member 5, also known as Binding Immunoglobulin Protein BIP or GRP-78), APLN (Apelin), LTB (Lymphotoxin-beta, also known as tumor necrosis factor C), CCL2 (C-C motif chemokine ligand 2, also known as monocyte chemoattractant protein-1), and DGAT2 (Diacylglycerol O-Acyltransferase). GAPDH gene and 18S rRNA were used for the normalization of qRT-PCR data of selected genes. Our validation results were in agreement with the microarray data and gave changes in the same order of magnitude (Figure 3).

### 3.2. Functional Enrichment Analysis

**Figure 4:**
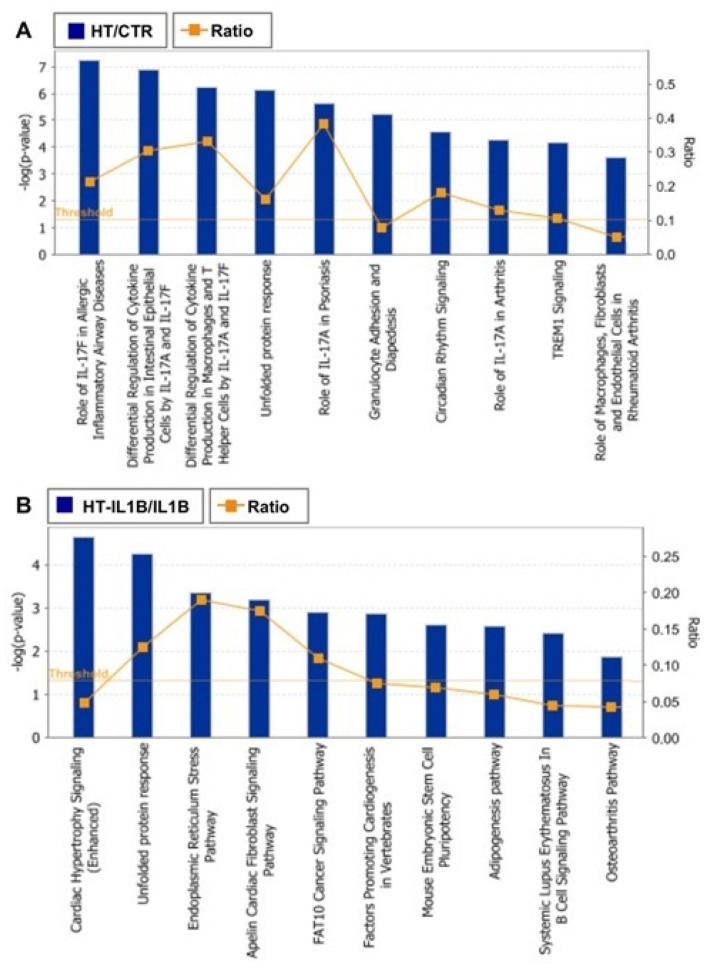
Top canonical pathways significantly modulated by HT at basal (**A**) and inflamed (**B**) conditions. Fisher’s exact test was used to calculate a *p*-value (shown as bars), determining the probability that the association between the genes in the data set and the canonical pathway is explained by chance. The ratio represents the number of differentially expressed genes in a given pathway divided by the total number of genes making up that canonical pathway.

To further define the biological processes altered in endothelial cells in response to olive oil polyphenol HT, functional enrichment analysis was performed on microarray data sets. Enrichment analysis on defined (canonical) pathways of IPA Knowledge Base provided significant over-represented pathways across the entire lists of DEGs in endothelial cells at basal and inflammatory conditions (Figure 4A,B and Tables S3 and S4).

**Figure 5:**
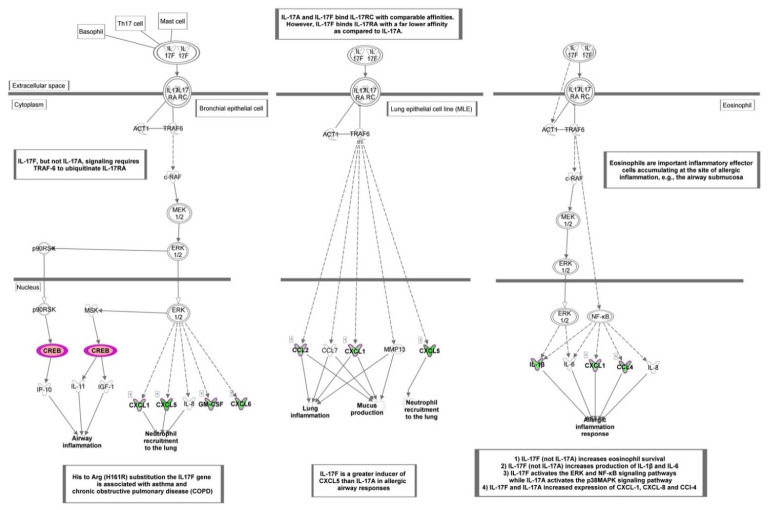
Canonical pathway representing “Role of IL-17F in allergic inflammatory airway diseases” obtained with Ingenuity Pathway Analysis (IPA) from the analysis of data set modulated by HT. Genes which expression was significantly modulated by HT are depicted together with others involved in the cascade (but not significantly modulated, depicted in gray). Complex or groups of genes are surrounded by a double line. Up-regulated genes are depicted in red, and down-regulated genes are depicted in green.

Among the top canonical pathways affected by HT in endothelial cells under resting conditions, “Role of IL-17F in allergic inflammatory airway diseases” was identified as the top one, with the highest –log (*p* value) (*p* < 5.62 × 10^−8^) (Figure 4A and Figure 5). This finding was related to other affected top canonical pathways, including: “Differential regulation of cytokine production in intestinal epithelial Cells by IL-17A and IL-17F” (*p* < 1.26 × 10^−7^), “Differential regulation of cytokine production in macrophages and T Helper cells by IL-17A and IL-17F” (*p* < 5.65 × 10^−7^), and “Role of IL-17A in psoriasis” (*p* < 2.31 × 10^−6^) (Figure 4A and Figure 5). These pathways contained mainly down-regulated genes related to inflammation and immunoregulation, such as cytokines (IL1B, and IL1A), chemokines (CCL2, CCL4, CXCL1, CXCL3, CXCL5, and CXCL6), and growth factors (CSF2, and CSF3) (Table S3).

**Figure 6:**
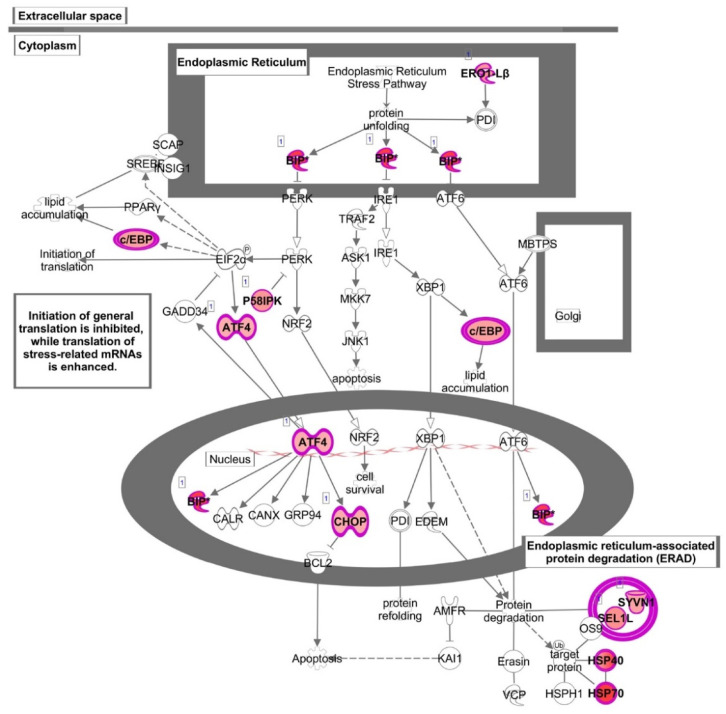
Canonical pathway representing “Unfolded protein response” obtained with Ingenuity Pathway Analysis (IPA) from the analysis of data set modulated by HT. Genes which expression was significantly modulated by HT are depicted together with others involved in the cascade (but not significantly modulated, depicted in gray). Complex or groups of genes are surrounded by a double line. Up-regulated genes are depicted in red.

“Unfolded protein response” (UPR) was also identified as one of the most affected canonical pathways (*p* < 5.73 × 10^−5^), with the highest number of up-regulated genes including transcription factors such as ATF4 (activating transcription factor 4), CEBPA (CCAAT enhancer binding protein alpha), DDIT3 (DNA damage inducible transcript 3), and chaperons such as HSPA5 (heat shock protein family A (Hsp70) member 5), as well as DNAJB9 (DnaJ heat shock protein family, Hsp40) and DNAJB3 (DnaJ heat shock protein family, Hsp40), ERO1B (endoplasmic reticulum oxidoreductase 1 beta), SEL1L (SEL1L adaptor subunit of ERAD E3 ubiquitin), and SYVN1 (synoviolin 1) involved in the control of protein folding and degradation (Figure 4 and Figure 6 and Table S3).

This pathway was also found among the most altered canonical pathways by HT in inflammatory conditions. Similar to baseline conditions, treatment with HT in the presence of IL-1β coincided with the up-regulation of genes as HSPA5 and DDIT3, as well as DNAJB9 and DNAJB3, ERO1B, SYVN1. In addition, HT-IL1B also up-regulated EIF2AK3 (eukaryotic translation initiation factor 2-alpha kinase 3), known as PERK. This transmembrane protein kinase has been defined as the central regulator of translational control during unfolded protein response and represents a linking node between NRF-2 function and endoplasmic reticulum (ER) stress. Accordingly, IPA analysis also detected “Endoplasmic reticulum stress” among the most representative pathways (*p* < 4.58 × 10^−4^) (Figure 4 and Table S4).

**Figure 7:**
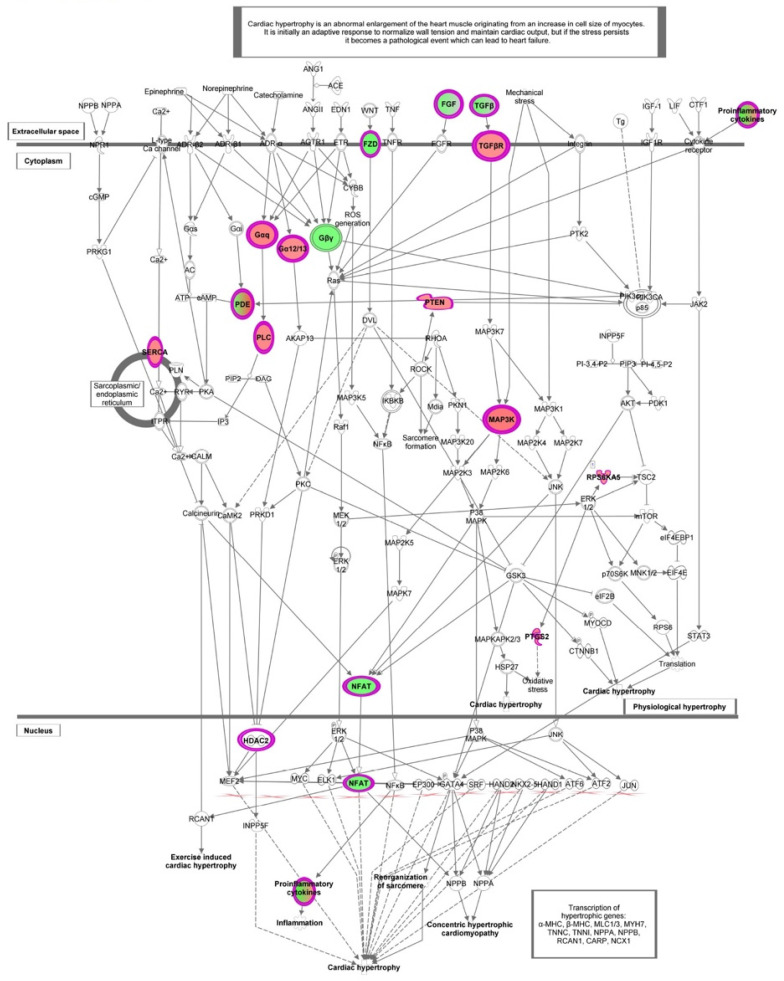
Canonical pathways representing “Cardiac hypertrophy signaling (enhanced)” obtained with Ingenuity Pathway Analysis (IPA) from the analysis of data set modulated by HT in IL-1β stimulated HUVECs. Genes which expression was significantly modulated by HT are depicted together with others involved in the cascade (but not significantly modulated, depicted in gray). Complex or groups of genes are surrounded by a double line. Up-regulated genes are depicted in red, and down-regulated genes are depicted in green.

In HT-IL1B, IPA showed “Cardiac hypertrophy signaling (enhanced)” as the top canonical pathway (*p* < 2.26 × 10^−5^) with altered expression of a remarkable number of genes affected by HT in inflamed endothelial cells (Figure 4 and Figure 7 and Table 4). In detail, HT induced the expression of genes including transcription regulators (HDAC4 and HDAC9), transporters (ATP2A2), kinases (BMPR2, RPS6KA5, MAP3K8), phosphatase (PTEN), and enzymes (FICD, GNA13, GNA14, PLCL2). Among the down-regulated genes associated with this canonical pathway, IPA revealed transcription regulator (NFATC1), growth factors (FGF16, FGF18, TGFB2 andTGFB3), cytokines (CD70 and LTB), and the enzyme PDE9A. G-protein coupled receptors were either up-regulated (FZD2) or down-regulated (FZD5).

Among the growth factors, the altered expression of TGFB2 and TGFB3 was also associated with other top canonical pathways as “Apelin cardiac fibroblast signaling pathway” (*p* < 6.59 × 10^−4^) and “FAT10 Cancer Signaling Pathway” (*p* < 1.29 × 10^−3^) (Figure 4 and Table S4), in addition to “Cardiac hypertrophy signaling (enhanced)”.

**Figure 8:**
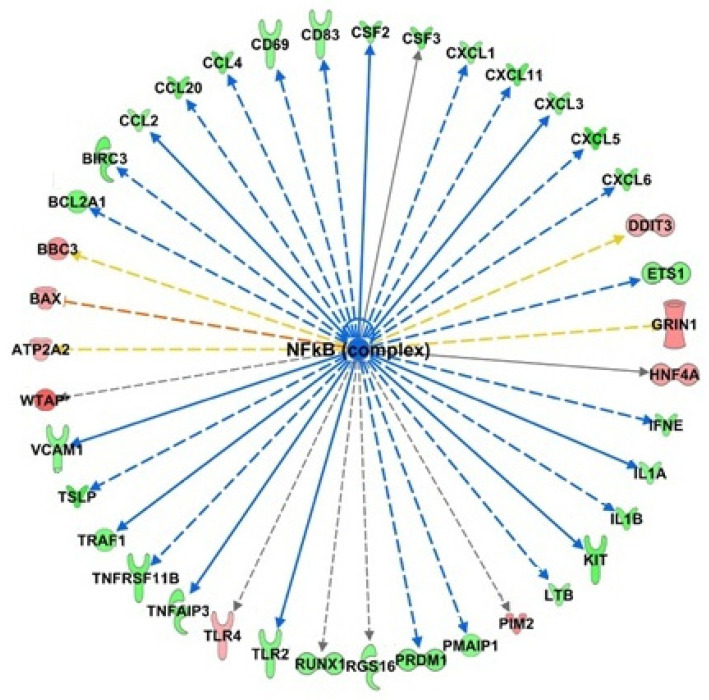
Identification of NF-κB as an upstream regulator of transcriptional regulation by HT. Bioinformatics analysis with IPA software predicts the inhibition of NF-κB as a regulator of down-regulated genes (depicted in green) and up-regulated (depicted in red).

To explore and characterize the molecular mechanisms underlying the effects of HT on endothelial cells, upstream regulator analysis was performed. The upstream regulator analysis infers the modulation of enzymes, kinases, and transcription regulators that could explain the observed gene expression changes. The cascade of events that lead to these gene expression alterations presumes the early involvement of these regulators. The analysis revealed that HT activated common regulators, under basal and inflamed conditions, related to the activation of UPR and endoplasmic reticulum stress pathways, such as XBP1, ERN1 (this gene encodes the transmembrane protein kinase inositol-requiring enzyme 1), and transcription regulators ATF6 with high z-score (+2.919, +2.54 and +2.189 for HT, and +3.466, +3.017, and +2.406 for HT-IL, respectively). These transcriptional regulators were related to the UPR and the endoplasmic reticulum stress pathway, and their activation. We also found that ATF4, an important transcription factor linked to UPR, was up-regulated by HT in endothelial cells at baseline, and it was predicted activated by HT under inflamed conditions using upstream regulator analysis. Moreover, the inhibition of common upstream regulators was also predicted, with TNF presenting the lowest z-score both in HT and HT-IL1B (−3.151 and −2.456, respectively). In HT-treated endothelial cells at resting conditions, NF-κB was the upstream regulator with the lowest z-score (z = 4.473). Among the downstream target genes regulated by NF-κB, the major significantly down-regulated in our data set were endothelial adhesion molecules, such as VCAM-1, and cytokines including IL-1A, IL1B, CCL2, CCL20, CCL4, CSF2, and CXCL3 involved in the leukocyte recruitment and inflammation (Figure 8).

## 4. Discussion

Our study reports the changes in the global gene expression of human endothelial cells exposed to HT under resting and proinflammatory conditions. The transcriptomic analysis reveals previously unsuspected genes and related gene pathways affected by the exposure of endothelial cells to HT, thus broadening our knowledge of its biological effects.

A considerable number of experimental studies and clinical intervention trials disclose health-promoting properties for HT and its derivatives obtained from olive oil consumption or intake of pure HT or olive polyphenols purified from olive mill wastewater [29]. However, the multiplicity of molecular mechanisms by which HT affects cardiovascular health remains insufficiently understood. To obtain a comprehensive overview of the biological processes modulated by olive oil polyphenols, including HT, whole-genome transcriptomic analyses have previously been performed on different kinds of human tissue but not always with consistent results [26,33]. After consumption of virgin olive oil rich in polyphenols, human PBMCs showed a shift towards a less deleterious inflammatory profile modulating the expression of genes involved in atherosclerosis, inflammation, and oxidative stress [10,11,27]. Nevertheless, studies examining the effects of HT on the whole-genome expression of endothelial cells, which are prominent targets for vasculo-protective effects of olive oil polyphenols, are still lacking.

The main objective of our analysis was to explore the effects of an acute supplementation of HT on changes in endothelial gene expression under resting and proinflammatory conditions. This latter goal was accomplished by comparing the gene expression profile of IL-1β-stimulated endothelial cells with those of cells treated with HT before IL-1β stimulation. Fixing rather stringent analytical conditions of fold change and significance (cut-offs > 1.5, *p*-values < 0.05), we identified 708 and 599 genes as differentially expressed by HT in endothelial cells under basal unstimulated and inflamed conditions, respectively. HT treatment led to a somewhat wide range of DEGs but with modest alterations in gene expression in terms of fold change. However, the cumulative effect of small changes in many genes combined may exhibit beneficial effects. This finding is consistent with previously reported actions of dietary-derived compounds usually characterized by the induction of a broad array of genes with modest changes in their intensity [12,34].

Enrichment analysis on defined (canonical) pathways of IPA Knowledge Base provided significant over-represented pathways affected by HT in endothelial cells at basal conditions. The most significant signaling pathways modulated by HT in endothelial cells include the “Role of IL-17F in Allergic Inflammatory Airway Diseases”, “Differential Regulation of Cytokine Production in Intestinal Epithelial Cells by IL-17A and IL-17F”, “Differential Regulation of Cytokine Production in Macrophages and T Helper Cells by IL-17A and IL-17F”, and “Role of IL-17A in Psoriasis” highlighting the influence of HT in IL-17 cytokine inflammatory pathways. As part of the adaptive and innate immunity, the IL-17 family is a critical component of the inflammatory response, acting on diverse cell types to induce the production of proinflammatory cytokines, chemokines, and prostaglandins [35]. Based on sequence homology, a total of six IL-17 members have been described, including IL-17A and IL-17F, able to bind the subunits of IL-17 receptor (IL-17 R), leading to the activation of transcription factors like NF-κB and AP-1, among others. IL-17A stimulated pathological changes associated with increased plaque instability, endothelial dysfunction and angiotensin II-induced hypertension. Meanwhile, IL-17A inhibition produced regression of atherosclerosis with lower expression of the chemokines, proinflammatory cytokines, and adhesion molecules, suggesting a modulatory role of IL-17A in vascular inflammation and related atherosclerosis [36]. IL-17 pathways, affected by HT, contained mainly down-regulated genes related to inflammation and immunoregulation, such as cytokines, chemokines, and growth factors. These genes contain recognition sequences for the redox-sensitive transcription factor NF-κB in their promoters [37]. Findings from our and other groups highlighted that HT reduces the expression of inflammatory chemokines and cytokines in endothelial cells at least in part through inhibiting the NF-κB signaling [19,23,38], supporting the role of NF-κB inactivation in the anti-inflammatory action of HT [6]. These findings are also consistent with transcriptomic data suggesting NF-κB as an upstream regulator in HT-treated endothelial cells. The targeting of the IL-17 pathway, here demonstrated, confirms and extends previous evidence, highlighting anti-inflammatory and vasculo-protective properties of HT [18,19,20,21,25]. Since IL-17 could also stimulate the trans-endothelial migration of neutrophils, our data suggest that HT could improve vascular function, thus preventing neutrophil recruitment characteristically associated with autoimmune diseases. Accordingly, some findings show that the secoiridoid HT derivative oleuropein decreases IL-17 expression and attenuates inflammatory damage in colonic samples from ulcerative colitis patients [39]. Moreover, a pilot trial provides evidence that olive polyphenols improve the cutaneous manifestations of the autoimmune disease psoriasis, characterized by critical endothelial–neutrophil interaction [40]. Whether HT exerts antipsoriatic effects by IL-17-mediated mechanism remains to be experimentally determined, but our data strongly support this hypothesis.

Functional enrichment analysis also identified “Unfolded protein response” as one of the most affected canonical pathways by HT in human endothelial cells, with many up-regulated genes, including transcription factors and chaperons involved in the control of endoplasmic reticulum homeostasis. The endoplasmic reticulum (ER) is a multifunctional cellular organelle that plays a paramount role in multiple physiological processes, including secretory and transmembrane protein folding and translocation, cellular Ca2+ uptake, storage and signaling, and cellular lipids production [41]. Several cardiovascular risk factors may interfere with ER function and affect its homeostasis with the ensuing accumulation of unfolded proteins in the ER lumen, thus favoring the establishment of ER stress condition [42]. Under ER stress, a signal transduction system known as unfolded protein response (UPR) is activated to restore protein homeostasis [43]. UPR can be activated through the involvement of three sensor pathways: PERK/ATF4 (protein kinase RNA-like ER kinase/activating transcription factor-4), IRE1/XBP1 (inositol requiring protein-1/X box binding protein 1), and ATF6 (activating transcription factor-6) [44]. The ER stress sensors PERK, IRE1, and ATF6 are usually maintained in their inactive forms through interaction with the immunoglobulin heavy chain-binding protein HSPA5 (BIP/GRP78) [44]. Our data reveals that HT treatment up-regulated the expression of ER chaperone protein HSPA5, a major modulator of UPR protective response, recently reported to play a key role in endothelial integrity [45,46]. Furthermore, HT up-regulated the expression of PERK/ATF4, known to promote a protective response during ER stress through an increase in nuclear factor erythroid 2-related factor 2 (NRF-2) expression and activation [47]. Our data suggest an influence by HT in ER stress and UPR, confirming and extending previous results by Zrelli et al. [48] that reported the ability of HT to induce a positive regulation of the antioxidant defense system in vascular endothelial cells through activation of NRF-2, which functionally translated in higher resistance to vascular injury. To activate the PERK/ATF4 signaling cascade, the upstream regulator in IPA analysis also predicted that HT could activate the other two arms of UPR, namely IRE/XBP1 and ATF6, since HT up-regulated the expression of downstream genes such as HSP proteins (DnaJ). Specifically, HT increased the expression of chaperone HSP40 (DnaJB9 and DnaJC3), which acts through ATPase activity stimulation of HSP70, also up-regulated by HT. Acquisition of stress tolerance by HSPs regulatory mechanisms was already described in mammalian cells pre-exposed to resveratrol [49] or blackberry-digested polyphenols [34]. Following our results, a transcriptomic analysis of colon cancer cells showed that rosemary polyphenols induced the activation of IRE1/XBP1 and PERK/ATF4 as a protective sensitization mechanism [50]. To our knowledge, this is the first study where HT was found to modulate key players of UPR in endothelial cells. This seems to be an adaptive mechanism in endothelial cells under control conditions and appears to improve the endothelial ability to respond effectively to stress induced by inflammatory stimuli. In line with our results, a robust and prolonged UPR induction has been associated with severe inflammatory responses. However, a mild UPR induction may be associated with protective effects in vascular endothelium and may serve as an attractive target for the development of new therapies against endothelial dysfunction [51]. In other cell systems, such as hepatocellular carcinoma, HT reduces ER stress induced by tunicamycin, suggesting that in conditions of strong ER stress, HT was able to modulate the response favorably [52]. In our data set, HERPUD1 was the most HT up-regulated gene in endothelial cells in both basal and IL-1β stimulated conditions. It is a key component of the multiprotein complex ER-associated protein degradation (ERAD), which controls the removal of misfolded proteins from the ER lumen, by modulating ER-derived quality control compartment assembly and facilitating the efficient degradation of unfolded proteins [53]. HERPUD1 interacts with the E3-ubiquitin ligases SYVN1 (HRD1) and SEL1L [54], also up-regulated by HT in endothelial cells. Experimental data demonstrate that, in diabetes animal models and related endothelial cell cultures, high glucose levels downregulate the expression of several factors related to ERAD signaling pathways with the resulting induction of endothelial dysfunction and diabetic retinopathy [55]. The observed increased expression of SYNV1 and HERPUD1 by HT could increase the ERAD ability to remove misfolded proteins, counteracting the loss of function of ERAD by vascular and metabolic risk factors. To the best of our knowledge, we propose for the first time ERAD as an HT target in promoting an adaptive response in endothelial cells.

Functional enrichment analysis revealed “Cardiac hypertrophy signaling (enhanced)” as the first canonical pathway associated with HT treatment in inflamed endothelial cells. The processes of growth (hypertrophy), angiogenesis, and metabolic plasticity are critically involved in the maintenance of cardiac homeostasis. Cardiac hypertrophy is classified as physiological when associated with normal cardiac function or as pathological when associated with cardiac dysfunction. The pathologic pathway involves calcineurin/nuclear factor of activated T-cells (NFAT) activation and fibrosis accumulation. NFAT is a family of transcription factors with a multidirectional regulatory function widely expressed in immune and nonimmune cells, including vascular endothelial cells. In quiescent cells, NFAT exists in the cytosol in a hyperphosphorylated state. Upon challenge with an inflammatory stimulus, calcineurin dephosphorylates NFAT, causing it to shuttle to the nucleus, where it increases the transcription of inflammatory genes [56]. NFAT signaling pathways play an essential role in regulating inflammatory mediators, extracellular matrix proteins, vascular permeability, and adhesion molecules involved in the progression of atherosclerosis [57]. Based on our microarray results, the expression of NFATC1 member was decreased by HT treatment in inflamed endothelial cells. This evidence suggests an additional potential mechanism by which HT counteracts vascular dysfunction induced by inflammatory triggers. In line with our findings, NFAT was inhibited by other polyphenols, including resveratrol, ferulic acid, and acteoside, a phenylethanoid glycoside that includes HT in its structure [58,59,60]. Finally, the canonical pathway “Cardiac Hypertrophy Signaling (Enhanced)” highlighted TGFB2 and TGFB3 genes reduced by HT treatment in inflamed endothelial cells. These growth factors have also been associated with other canonical pathways affected by HT, including “Apelin Cardiac Fibroblast Signaling Pathway” and “FAT10 Cancer Signaling Pathway”. TGFβ superfamily includes three TGFβ isoforms (TGFβ1, TGFβ2, and TGFβ3) involved in a wide range of diverse functions both in physiological and pathological processes [61]. TGF-β is a well-known inducer of endothelial-to-mesenchymal transition, representing a critical link in the complex interactions between inflammatory stress and endothelial dysfunction. It is involved in a variety of biological processes that drive the formation of fibrosis, accompanying many disease states [62,63]. Our microarray data suggest, for the first time, that HT treatment reduced the IL-1β up-regulated expression of TGF-β2 and β3 in endothelial cells, thus revealing another mechanistic explanation for the anti-inflammatory and vascular beneficial properties attributed to HT, including a potential antifibrotic effect. In accordance with our findings, literature evidence indicated a therapeutic potential of polyphenols in cardiac fibrosis, mediated by down-regulation of TGF-β genes [64], and reported the inhibitory role of HT on breast cancer stem cells by targeting epithelial-to-mesenchymal transition and TGF-β signaling pathways [65].

Overall, our transcriptomic analysis allowed us to pinpoint immunological, inflammatory, proliferative and metabolic-related pathways as the most affected by HT in endothelial cells. Regarding the biological interpretation of microarray data, we hypothesize that HT reduces the endothelial response under proatherogenic stimuli through the modification of homeostatic cellular processes such as UPR to counteract the ER stress and increase the antioxidant defenses, thus preventing apoptosis and inflammation. HT also decreases the activation of proinflammatory transcription factors such as NF-kB and NFAT directly or by reducing the production of factors (cytokines, chemokines, growth factors) that induce their activation. Moreover, HT reduces the expression of immuno-activating and proinflammatory factors, which are involved in the initial stages but also in the progression and complication of plaque and in hypertrophy and neointima formation. The endothelium in the vascular bed interacts with thousands of regulators and substances, including nutrients and non-nutritive compounds. Studies that analyzed the effect of HT as a pure molecule or as extra virgin olive oil polyphenolic extract showed a synergistic vasculo-protective action between different phenolic compounds [29]. Furthermore, our previous study showed that the circulating metabolites obtained from the serum of healthy subjects who took extra virgin olive oil rich in polyphenols improved endothelial function, suggesting a vascular-protective role in vivo [21]. The unbiased identification of novel genes regulated by HT improves our understanding of mechanisms by which olive oil polyphenols prevent and attenuate inflammatory diseases via the regulation of endothelial cell genomic response and discovers new unsuspected genes and associated pathways to be enquired as potential contributors to the interindividual variation in response to consumption of plant food bioactives. The use of whole transcriptional profiling represents a powerful tool in nutrigenomic studies. It allows for unbiased measurement not marred by a priori hypotheses of the active changes in gene expression before and after the addition or administration of a nutrient or a nutrient metabolite to cultured cells or animal models of disease. The results of such studies allow us to inventory and catalogue nutrient-responsive genes disclosing all the associated plausible nodes of signaling pathways. Up to now, the majority of in vivo human nutrigenomic studies have analyzed PBMCs as a feasible human-derived cell type [26]. However, being nutrigenomic effects potentially tissue-dependent the gene expression measurements in human tissues other than PBMCs, such as in endothelial cells, is becoming mandatory for a more comprehensive evaluation of potential therapeutic of a nutrient. The results of our analysis have broken the ground for the identification of genes (and related pathways) underlying the potential health effects of HT. This deepening in the HT mechanisms of action will allow us to identify novel unsuspected genes whose polymorphisms might be investigated in humans to better explain some aspects of the inter-individual variability in response to extra virgin olive oil consumption. This means that our data will allow us to configure nutrigenetic studies aiming to identify HT responsive genotypes so that the antioxidants intake may be optimized to determine the maximal therapeutic effects reducing the risk of adverse unforeseen side effects.

These findings derive from transcriptomics analyses; therefore, further studies are needed to evaluate protein levels and associated signaling mechanisms using specific approaches. Moreover, as our study used a human cell culture model, these data require appropriate confirmation in vivo before being transferred to humans.

## 5. Conclusions

In conclusion, the data presented here yield novel information on vascular health effects linked to HT and olive oil polyphenol intake. They demonstrate that exposure of human endothelial cells to HT results in specific and multiple changes in gene expression at basal and inflammatory conditions that may contribute to preventing endothelial dysfunction and inflammation. Several pathways here shown to be regulated by HT are novel and suggest promising reinterpretations of the health-promoting potential of HT in inflammatory, dysmetabolic, and (auto)immune diseases.

## Figures and Tables

**Table 1:** Primers sequence for qRT-PCR.

Gene Name	Accession Number	Forward Primer	Reverse Primer	Size (bp)
HERPUD1	NM_014685.4	5′- TATGGGTGGCTTCAGCTTTC-3′	5′-GGCTCCAGGATTAACAACCA-3′	228
DNAJB9	NM_012328.3	5′-TCGGAGGGTGCAGGATATTA-3′	5′-AGCATCCGGGCTCTTATTTT-3′	212
HSPA5	NM_005347.5	5′-TGCAGCAGGACATCAAGTTC-3′	5′-AGTTCCAGCGTCTTTGGTTG-3′	245
DGAT2	NM_032564.5	5′-GCTGACCTGGTTCCCATCTA-3′	5′-CAGGTGTCGGAGGAGAAGAG-3′	164
FGF18	NM_003862.3	5′-GGACCAGTGGGAAACACATC-3′	5′-CAGGGCCGTGTAGTTGTTCT-3′	245
APLN	NM_017413.5	5′-CCAAGGAGCAGCATGAATCT-3′	5′-GAAAGGCATGGGTCCCTTAT-3′	243
LTB	NM_002341.2	5′-AGGAGCCACTTCTCTGGTGA-3′	5′-CAGCTTCTGAAACCCCAGTC-3′	151
CCL2	NM_002982.3	5′-CCCCAGTCACCTGCTGTTAT-3	5′-TCCTGAACCCACTTCTGCTT-3′	166
CXCL5	NM_002994.5	5′-CCACTATGAGCCTCCTGTCC-3′	5′-CTATGGCGAACACTTGCAGA-3′	219
CXCL11	NM_005409.5	5′-AGAGGACGCTGTCTTTGCAT-3′	5′-TAAGCCTTGCTTGCTTCGAT-3′	175
GAPDH	NM_002046.3	5′-ATCACTGCCACCCAGAAGAC-3′	5′-TTCTAGACGGCAGGTCAGGT-3′	210
18S rRNA	NR_003286.2	5′-AAACGGCTACCACATCCAAG-3′	5′-CCTCCAATGGATCCTCGTTA-3′	155

The quantifications were performed using the efficiency-adjusted ΔΔCT method (CFX Manager). Results are expressed as fold increase relative to unstimulated control (made = 1).

**Table 2:** Gene expression profiling of HT treated endothelial cells by IPA analysis.

Fold Change	HT/CTR			HT-IL1B/IL1B		
	Total	Numbers	Regulation	Total	Numbers	Regulation
>2.0	71	44	up	45	33	up
		27	down		12	down
1.5<~<2.0	398	249	up	390	243	up
		149	down		147	down
Total	469	293	up	435	276	up
		176	down		159	down

**Table 3:** The top 10 up-regulated or down-regulated genes by HT in endothelial cells at basal condition (HT/CTR).

HT/CTR							
Symbol	Entrez Gene Name	Function	Fold Change	Symbol	Entrez Gene Name	Function	Fold Change
HERPUD1	homocysteine inducible ER protein with ubiquitin-like domain 1	regulator of protein degradation	6.603	CXCL11	C-X-C motif chemokine ligand 11	chemotactic protein	−2.181
DNAJB9	DnaJ heat shock protein family (Hsp40) member B9	co-chaperone	4.179	CCDC191	coiled-coil domain containing 191	uncharacterized	−2.215
CDH15	cadherin 15	cell adhesion protein	3.803	SNHG8	small nucleolar RNA host gene 8	long noncoding RNA	−2.232
SDF2L1	stromal cell-derived factor 2 like 1	chaperone binding	3.617	LOC730101	uncharacterized LOC730101	uncharacterized	−2.318
HSPA5	heat shock protein family A (Hsp70) member 5	chaperone	3.611	FGF18	fibroblast growth factor 18	growth factor	−2.344
WTAP	WT1 associated protein	splicing regulator	2.992	TSLP	thymic stromal lymphopoietin	hemopoietic cytokine	−2.562
H1-5	H1.5 linker histone. cluster member	chromatin DNA binding	2.969	CXCL5	C-X-C motif chemokine ligand 5	Chemotactic protein	−2.708
NMRAL2P	NmrA-like redox sensor 2. Pseudo gene	uncharacterized	2.907	ZNF33B	zinc finger protein 33B	transcription regulator	−2.742
NEUROG3	neurogenin 3	transcription regulator	2.847	CDADC1	cytidine and dCMP deaminase domain containing 1	DNA cytosine deamination	−2.758
FICD	FIC domain containing	Nucleotidyl transferase	2.740	ZNF594	zinc finger protein 594	transcription regulator	−3.310

**Table 4:** The top 10 up-regulated or down-regulated genes by HT in endothelial cells at stimulated conditions (HT-IL1B/IL1B).

HT-IL1B/IL1B							
Symbol	Entrez Gene Name	Function	Fold Change	Symbol	Entrez Gene Name	Function	Fold Change
HERPUD1	homocysteine inducible ER protein with ubiquitin-like domain 1	regulator of protein degradation	5.383	IP6K2	inositol hexakisphosphate kinase 2	kinase	−2.024
DNAJB9	DnaJ heat shock protein family (Hsp40) member B9	co-chaperone	4.232	ZNF33B	zinc finger protein 33B	transcription regulator	−2.026
MAT2A	methionine adenosyltransferase 2A	transferase	2.866	APLN	Apelin	protein binding	−2.233
ZNF658	zinc finger protein 658	transcription regulator	2.793	LTB	lymphotoxin beta	cytokine	−2.238
HSPA5	heat shock protein family A (Hsp70) member 5	chaperone	2.754	RBM12B	RNA binding motif protein 12B	RNA binding	−2.238
ALPK1	alpha kinase 1	kinase	2.668	RGS16	regulator of G protein signaling 16	enzyme	−2.270
ERO1B	endoplasmic reticulum oxidoreductase 1 beta	oxidoreductase	2.647	SRA1	steroid receptor RNA activator 1	transcription regulator	−2.340
FDXACB1	ferredoxin-fold anticodon binding domain containing 1	uncharacterized	2.592	ZNF594	zinc finger protein 594	transcription regulator	−2.586
SLC7A11	solute carrier family 7 member 11	transporter	2.559	DGAT2	diacylglycerol O-acyltransferase 2	acyltransferase	−2.648
SLC10A7	solute carrier family 10 member 7	transporter	2.465	APOL6	apolipoprotein L6	lipid binding	−2.824

## Supplementary Materials

The following are available online at https://www.mdpi.com/article/10.3390/nu13113990/s1, Table S1: Deregulated genes by HT in endothelial cells at basal conditions (HT/CTR), Table S2: Deregulated genes by HT in endothelial cells at inflamed conditions (HT-IL1B/IL1B), Table S3: Canonical pathway significantly modulated by HT in endothelial cells at basal conditions (HT/CTR), Table S4: Canonical pathways significantly modulated by HT in endothelial cells at inflamed conditions (HT-IL1B/IL1B).
